# Topographic Quadrant Analysis of Peripapillary Superficial Microvasculature in Optic Disc Drusen

**DOI:** 10.3389/fneur.2021.666359

**Published:** 2021-05-19

**Authors:** Yan Yan, Xiao Zhou, Zhongdi Chu, Laurel Stell, Mohammad Ali Shariati, Ruikang K. Wang, Yaping Joyce Liao

**Affiliations:** ^1^Department of Ophthalmology, Stanford University School of Medicine, Stanford, CA, United States; ^2^Department of Ophthalmology, Renji Hospital, School of Medicine, Shanghai Jiao Tong University, Shanghai, China; ^3^Department of Bioengineering, University of Washington, Seattle, WA, United States; ^4^Department of Biomedical Data Science, Stanford University School of Medicine, Stanford, CA, United States; ^5^Department of Ophthalmology, University of Washington, Seattle, WA, United States; ^6^Department of Neurology, Stanford University School of Medicine, Stanford, CA, United States

**Keywords:** optic disc drusen, optical coherence tomography, optical coherence tomography angiography, vessel area density, retinal nerve fiber layer, visual field

## Abstract

**Background:** Limited information is known about the topographic effect of optic disc drusen (ODD) on peripapillary retinal nerve fibers and microvasculature.

**Objective:** This study aims to understand the structural and functional impact of ODD in different quadrants of the optic disc.

**Methods:** We performed a retrospective case-control study of 22 ODD patients (34 eyes) and 26 controls (33 eyes) to compare optical coherence tomography (OCT) retinal nerve fiber layer (RNFL), OCT angiography (OCTA), and corresponding static perimetry mean deviation (MD) calculated using the modified Garway-Heath map in different quadrants of the optic disc. OCTA was analyzed using custom MATLAB script to measure six parameters in a peripapillary annulus with large vessel removal: vessel area density (VAD), vessel skeleton density (VSD), vessel perimeter index (VPI), vessel complexity index (VCI), flux, and vessel diameter index (VDI).

**Results:** Quadrant analysis revealed that OCTA VAD and VCI were significantly decreased in superior, nasal, and inferior but not temporal quadrant. RNFL, VSD, and VPI were significantly impacted only in the superior and nasal quadrants. Corresponding visual field MDs in all ODD eyes were not different in the four quadrants, although eyes with MD equal or worse than −5 dB (32%) had worst visual field corresponding to the superior quadrant of the optic disc (inferior arcuate visual field). Structure-structure comparison of OCT and OCTA showed high correlation of RNFL with multiple OCTA measurements in the superior, nasal, and inferior quadrants but not temporal quadrant. Structure-function analysis revealed significant correlation of VAD and VCI and visual field MD in every quadrant, but RNFL was only significantly correlated in the superior and inferior quadrants.

**Conclusions:** Peripapillary VAD and VCI are decreased in more quadrants than RNFL, supporting the clinical utility of performing OCTA in addition to OCT. Consistent with the most common locations of ODD, five OCT/OCTA measurements (VAD, VCI, RNFL, VSD, VPI) are decreased in the superior and nasal quadrants. OCT/OCTA measurements were significantly impacted in contrast to the relatively mild effect on corresponding visual field MD, consistent with the idea that a decrease in *objective* structural and vascular measurements occurs without parallel change in *subjective* visual function in ODD.

## Introduction

Optic disc drusen (ODD) is an optic neuropathy characterized by semitranslucent calcified yellowish depositions in the anterior, unmyelinated optic nerve (1). They are found in up to 2.0% of the general population (2). ODD are often noted incidentally as part of an eye exam, and patients may have no visual symptoms. ODD are most commonly found in the nasal side of the disc in idiopathic as well as syndromic ODD (3–5). Pathologically, ODD is characterized by deposition of calcified bodies of various sizes ranging from 5 to 1,000 μm, calcified mitochondria in intact axons, as well as in the extracellular space, and abnormal vasculature with enlarged perivascular space (6).

Visual field can be normal in ODD eyes or present as arcuate visual field defect or constricted visual field (2, 7–9). Visual field loss in ODD can be slowly progressive, most commonly affecting the inferonasal and inferotemporal quadrants (3, 10, 11). Visual field defect is worse in those with superficial ODD (2). The most common cause of sudden vision loss in ODD is non-arteritic anterior ischemic optic neuropathy (ODD-AION) (12–14), and this condition is due to vascular compromise at the optic nerve head, possibly due to the compressive effect of the ODD on the optic nerve axons, vasculature, and surrounding structures. Vascular compression or altered autoregulation also leads to increased risk of vascular complications, such as non-arteritic anterior ischemic optic neuropathy, central retinal artery occlusion, central retinal vein occlusion, and peripapillary choroidal neovascularization (2).

Studies comparing modern non-invasive ophthalmic imaging led to the recommendation that enhanced depth imaging optical coherence tomography (EDI-OCT) is the gold standard in the diagnosis of ODD (15, 16). Previous OCT studies also revealed that ODD patients had significantly thinned retinal nerve fiber layer (RNFL) and ganglion cell complex (GCC) for the average RNFL/GCC thickness or in certain quadrants (17, 18). More recently, OCT angiography (OCTA) has been used to detect microvascular changes in the peripapillary and macular areas in different optic neuropathies (19) including ODD (20). Previous studies found that eyes with ODD had focal microvascular attenuation corresponding to the drusen (21–23).

Based on *en face* imaging and OCT, ODD are most commonly located in the nasal and superior optic disc (5), which means there are topographic differences in the involvement of the retinal ganglion cell axons at the unmyelinated, anterior optic nerve, and, presumably, their surrounding microvasculature. This location corresponds to the most common visual field deficits in the inferonasal and inferotemporal quadrants (2). However, limited information is known about the topographic impact of ODD on peripapillary OCT, OCTA, and visual function. The reason for the nasal and superior localization of ODD is unclear, but previous publications have shown that the seesaw-like distortions of the optic disc as a result of horizontal eye movement, especially with adduction, is present in ODD eyes and likely exert repetitive shearing or strains on the peripapillary tissue, potentially leading to local axonal and vascular impact (24). OCT studies have demonstrated that RNFL thickness is significantly decreased in all quadrants except in the temporal quadrant in eyes with ODD (17).

The purpose of this study was to perform quadrant analysis of visual field mean deviation and peripapillary OCT and OCTA based on modified Garway-Heath map. Such analysis will determine whether there is differential impact of ODD on peripapillary OCT and OCTA measurements in different quadrants and whether there is high structure-function and structure-structure correlations. Such analysis will help determine whether visual field mean deviation, OCT, and OCTA measurements are differentially affected in different quadrants in this condition with clear topographic differences.

## Patients and Methods

### Study Design and Participants

We performed a retrospective cross-sectional OCT and OCTA study of consecutive patients with ODD who were evaluated at the Byers Eye Institute at Stanford University Medical Center between January 2016 and April 2020, qualified for the study based on inclusion and exclusion criteria, and had high-quality spectral-domain OCT and OCTA using the same machine (AngioPlex, Model 5000; Carl Zeiss Meditec In., Germany). The study was approved by the Institutional Review Board of Stanford University and adhered to the Declaration of Helsinki and the Health Insurance Portability and Accountability Act.

We recruited 22 patients (34 eyes) with ODD. All subjects had comprehensive ophthalmic examination, including best corrected visual acuity (BCVA) using the Snellen chart to calculate the logarithm of reciprocal decimal visual acuity (logMAR VA), refraction, intraocular pressure, and fundus examination. Patients were included for the study if the diagnosis of ODD was confirmed by color fundus imaging, fundus autofluorescence, and EDI-OCT. We also recruited age- and sex-matched controls, 33 eyes from 26 healthy controls for comparison. The control subjects had BCVA of equal or better than the logMAR VA 0.2, normal intraocular pressure, color vision, normal ocular fundus, no visual field defect, and normal RNFL or GCC thickness. *Superficial ODD* is defined as visible ODD on the optic disc by ophthalmoscopy, appearing as bright refractile depositions, and *buried ODD* are ODD that are not visible on ophthalmoscopy (25, 26). We excluded all patients with history of optic neuropathy other than ODD and those with ophthalmic, neurological, or systemic diseases that may affect the measurements of OCT and OCTA.

### OCT and OCTA Acquisition, Processing, and Quantification

**Figure 1:**
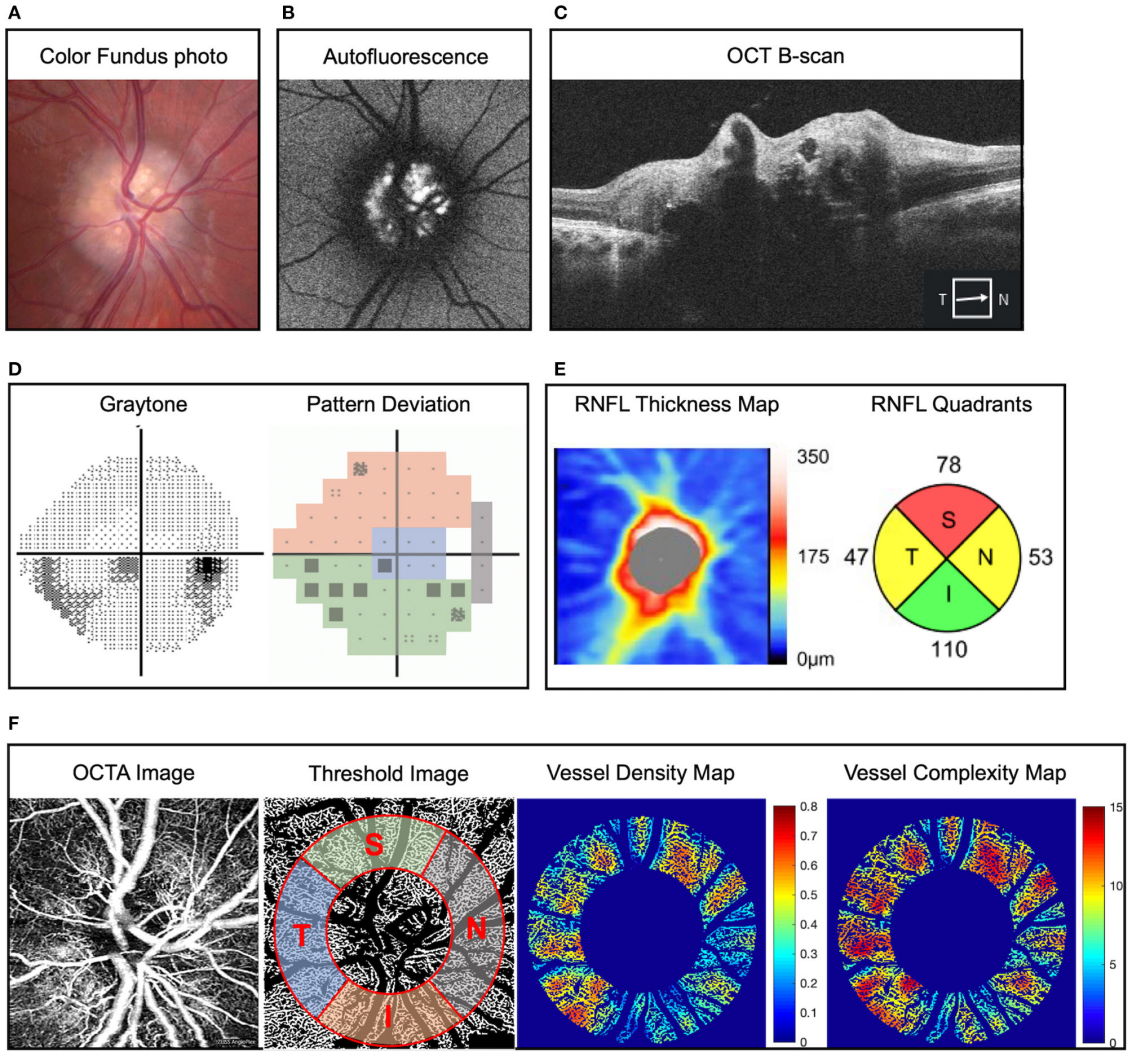
Representative example of optic disc drusen (ODD) and right eye mild inferior visual field defect in a 22-year-old man. **(A)** Color fundus imaging showing full optic disc with refractile depositions in the optic disc and slightly blurred margin (right eye). **(B)** Autofluorescence imaging showing hyperautofluorescence of the optic disc typical of ODD. **(C)** Optic coherence tomography (OCT) B-scan reveals characteristic signal poor core with bright margins typical of ODD. **(D)** Static perimetry reveals inferior visual field defect in the right eye, and pattern deviation map demonstrates the modified Garway-Heath map of visual field. **(E)** Retinal nerve fiber layer (RNFL) thickness map on OCT showing superotemporal and nasal RNFL defect in the right eye. **(F)** Superficial capillary plexus of optic disc OCT angiography (OCTA) (*from left to right*) original image, threshold image with large vessel removal, annulus region of interest, and demarcation of the four quadrants according to the modified Garway-Heath map for visual field representation, vessel density heat map, and vessel complexity heat map. Vessel area density and vessel complexity index are calculated and represent area occupied by vessel divided by total area and branching complexity of the perfused vasculature, respectively (27). For ease of comparison, these values are shown on a linear color scale (heat map) from blue to red, which represents low to high vessel densities or low to high vessel complexities. VAD heat map scale is 0 to 0.8, and VCI heat map scale is 0 to 15. OCT, optical coherence tomography; OCTA, optical coherence tomography angiography; RNFL, retinal nerve fiber layer.

OCT and OCTA images were acquired using Cirrus HD-OCT (AngioPlex, Model 5000; Carl Zeiss Meditec In., Germany). We performed the Optic Disc Cube scan pattern acquiring 200 horizontal scan lines each composed of 200 A-scans. The thickness of RNFL was measured on a circle with 3.46 mm diameter centered on the optic disc. The Cirrus HD OCT optic disc protocol generates a map with quadrant RNFL thickness (superior, nasal, inferior, and temporal) (Figure 1E). The 3 × 3 mm^2^ square scans of the optic disc were obtained using the FastTrac eye tracking system for OCTA imaging. Algorithms for optical microangiography (OMAG)-based automatic segmentation of the raw OCTA data were used to get superficial retinal layer (SRL) *en face* peripapillary OCTA image (28). Only images with signal strength index > 7 were used for analysis. We use validated customized quantification software (coded with MATLAB R2016a; MathWorks, Natick, MA) to quantify OCTA images based on the modification of previous algorithm, and six vessel parameters of the peripapillary retina were obtained the same way as the previous study, including vessel area density (VAD), vessel skeleton density (VSD), vessel perimeter index (VPI), vessel complexity index (VCI), vessel diameter index (VDI), and flux index (20, 27, 29). We removed the large vessels from the images and analyzed an annulus with an outer diameter of 2.75 mm and an inner diameter of 1.5 mm centered around the disc. The OCTA parameters in superior, nasal, inferior, and temporal quadrants according to modified Garway-Heath map were measured using the quantification software as shown in Figure 1F.

### Visual Field Assessment

Visual field test was evaluated with Humphrey Field Analyzer (Carl Zeiss Meditech, Inc, Dublin, CA) using the standard 24-2 Swedish Interactive Thresholding Algorithm fast strategy. The mean deviation (MD) was automatically calculated by the Humphrey Field Analyzer. Sectorial division of peripapillary region including nasal, inferior, superior and temporal area was performed using Garway-Heath map regionalization (Figure 1D) (30). We excluded unreliable visual field tests, which were defined as fixation loss >20% or false-positive or false-negative error rates >20%.

### Statistical Analysis

The data were analyzed by SPSS version 23.0 (SPSS Inc., Chicago, IL, USA) and R (R Foundation for Statistical Computing, Vienna, Austria). Quantitative continuous variables were calculated as mean ± standard error (SE) or median (95% confidence interval). A two-tailed Mann-Whitney *U* test was used to compare the means of two groups of continuous variables. The frequencies of categorical variables were compared using Chi-square test. Kruskal-Wallis *H* test was performed to determine if there are statistically significant differences among more than two groups of independent variables. Spearman correlation test was performed to determine the correlation between OCT, OCTA, and visual field MD parameters. The correlation coefficients between 0 and 0.3 indicate weak correlation, between 0.3 and 0.7 indicate moderate correlation, and between 0.7 and 1 indicate high correlation. A value of *P* <0.05 was considered statistically significant.

## Results

### Demographic and Clinical Characteristics of Patients With Optic Disc Drusen

We performed comparison of static perimetry, OCT, and OCTA in 34 eyes of 22 ODD patients (female:male = 2.14:1) and 33 eyes of 26 controls (Table 1). Of the 22 ODD patients, 12 were bilateral and 10 were unilateral. Twelve eyes (35.3%) had buried drusen, and 22 (64.7%) had superficial ODD. Eyes with ODD and control groups did not differ by age, sex, and LogMAR VA. The mean age of patients with ODD was 43.3 years (range: 13 to 76 years), and controls were 48 years (range: 15 to 77 years). Mean age of patients with buried drusen was 36.8 years (median: 32 years, range: 13 to 65 years), and those with superficial ODD were older with mean age of 46.8 years (median: 48 years, range: 16 to 76 years). On static perimetry, eyes with ODD had a significantly worse MD compared with that of controls [ODD: −4.37 ± 1.00 dB (range: −23.11 to 0.18 dB), control: −0.21 ± 0.13 dB (range: −1.66 to 1.54 dB); *P* < 0.0001]. Despite this significant difference, the majority of ODD eyes had relatively good average MD (68% of eyes better than −5 dB and 93% better than −10 dB). Eyes with buried ODD had relatively better visual field MD of −1.2 ± 0.17 dB, and eyes with superficial ODD had worse visual field MD of −5.58 ± 1.09 dB. The difference of average MD between normal controls and buried ODD was statistically significant (*P* = 0.006), so was the difference between buried and superficial ODD (*P* = 0.006).

**Table 1:** The demographics parameters of controls and patients (eyes) with optic disc drusen.

**Variable**	**Control (*n* = 33 eyes)**	**ODD (*n* = 34 eyes)**	***P*-value^b^**
Age (year)	48.0 ± 3.0	43.3 ± 3.3	0.273
**Sex [*****n*** **(%)]**			0.153
Female	14 (53.8)	15 (68.2)	
Male	12 (46.2)	7 (31.8)	
LogMAR VA	0.01 ± 0.01	0.03 ± 0.01	0.134
VF MD (dB)	−0.21 ± 0.13	−4.37 ± 1.00^a^	**<0.0001**

*LogMAR, logarithm of the minimum angle of resolution; MD, mean deviation; ODD, optic disc drusen; VA, visual acuity; VF, visual field*.

*Data are expressed as mean ± standard error except sex. Values were compared by Mann–Whitney test. Sex was compared by Fisher's exact test*.

a *Calculated from 29 eyes*.

b *P < 0.05 was considered to be statistically significant. Significant value is shown in bold*.

Figure 1 shows ophthalmic imaging and static perimetry of the right eye of a 22-year-old Caucasian man with bilateral ODD. On color fundus imaging, there was a typical lumpy bumpy appearance of the entire optic disc with highly refractile deposits and blurred disc margin. On fundus autofluorescence imaging, there was hyperautofluorescence of the entire optic disc, which corresponded with ODD of variable sizes on OCT B-scan (Figures 1A–C). Peripapillary OCT analysis revealed reduced average RNFL thickness as well as reduced sectoral RNFL thickness of <1%-tile of age-matched controls in the superior quadrant and <5%-tile of age-matched controls in the nasal and temporal quadrants (Figure 1E). To analyze the peripapillary superficial capillary plexus OCTA images, the original images were processed to remove large vessels and overlayed with an annulus region-of-interest (see Methods). We used a custom MATLAB script to calculate six OCTA parameters (27) for each quadrant. Figure 1 shows that the eye with ODD had reduced VAD (area occupied by blood vessels divided by total area) and decreased VCI (branching complexity of the perfused vasculature). These changes (heat map in Figure 1F) correspond with reduced visual field on static perimetry (Figure 1D).

### Significant Reduction of OCTA Measurements in More Quadrants Than OCT RNFL

We compared the average peripapillary OCT RNFL and superficial capillary plexus OCTA parameters for all 34 ODD eyes and 32 control eyes. Compared with controls, ODD eyes had significantly thinner RNFL by 12 μm (control: 94.9 ± 1.6 μm, ODD: 82.8 ± 4.2 μm, difference 13%, *P* < 0.0001). On OCTA, ODD eyes also had significantly lower measurements in *VAD* (control: 0.48 ± 0.01, ODD: 0.44 ± 0.01, difference 8%, *P* = 0.007), *VSD* (control: 0.18 ± 0.00, ODD: 0.17 ± 0.00, difference 6%, *P* = 0.017), *VPI* (control: 0.39 ± 0.00, ODD: 0.36 ± 0.01, difference 8%, *P* = 0.026), and *VCI* (control: 2,193.5 ± 25.3, ODD: 2,025.3 ± 44.2, difference 8%, *P* = 0.010). There was no difference in VDI (difference 0%, *P* = 0.331) or flux (difference 5%, *P* = 0.110) between controls and ODD groups. Thus, OCT RNFL and OCTA VAD, VSD, VPI, and VCI were the five most useful measurements.

Given ODD is more common in the nasal and superior disc, we analyzed OCT and OCTA measurements in four quadrants of the optic disc and calculated corresponding visual field MD based on modified Garway-Heath map (Figure 1). Quadrant analysis revealed that OCT RNFL thickness was significantly lower in the superior and nasal quadrants but not in the inferior and temporal quadrants in ODD eyes compared with controls (Table 2). Of the four OCTA measurements that were significantly different between ODD and control groups, VCI was the only one that was significantly lower in all quadrants, while VAD—the most commonly calculated measurement in commercial OCTA machines—was significantly reduced in superior, nasal, and inferior but not temporal quadrants (Table 2). VSD and VPI were significantly different between ODD and control groups in superior and nasal quadrants. Although the difference of average flux was not significant, flux measurement in the *nasal* quadrant was significantly lower in the ODD group compared with controls. There was no difference in VDI in all four quadrants.

**Table 2:** Differences in parapapillary OCT and OCTA parameters by quadrants between controls and eyes with optic disc drusen.

**Parameter**	**Control group (*****n*** **=** **33)**				**ODD Group (*****n*** **=** **34)**				***P*****-value^a^**			
	**Superior**	**Nasal**	**Inferior**	**Temporal**	**Superior**	**Nasal**	**Inferior**	**Temporal**	**Superior**	**Nasal**	**Inferior**	**Temporal**
RNFL (μm)	118.4 ± 2.7 (119.0, 112.8~123.9)	72.7 ± 1.7 (72.0, 69.2~76.2)	123.2 ± 2.1 (122.0, 118.9~127.6)	66.9 ± 1.9 (65.0, 63.0~70.8)	94.3 ± 7.3 (90.0, 79.5~109.1)	59.2 ± 2.5 (54.5, 54.1~64.2)	110.9 ± 5.8 (115.5, 99.2~122.7)	64.8 ± 4.0 (64.0, 56.7~72.9)	**<0.0001**	**<0.0001**	0.086	0.363
Disc VAD	0.48 ± 0.01 (0.49, 0.47~0.49)	0.46 ± 0.01 (0.47, 0.44~0.47)	0.47 ± 0.00 (0.47, 0.46~0.48)	0.50 ± 0.01 (0.49, 0.48~0.51)	0.43 ± 0.01 (0.45, 0.41~0.46)	0.41 ± 0.01 (0.43, 0.39~0.44)	0.43 ± 0.01 (0.45, 0.40~0.46)	0.49 ± 0.01 (0.49, 0.47~0.51)	** <0.0001**	** <0.0001**	**0.025**	0.975
VSD	0.18 ± 0.00 (0.18, 0.18~0.19)	0.18 ± 0.00 (0.18, 0.18~0.19)	0.17 ± 0.00 (0.16, 0.16~0.18)	0.19 ± 0.00 (0.19, 0.19~0.20)	0.17 ± 0.00 (0.17, 0.16~0.17)	0.16 ± 0.00 (0.17, 0.15~0.17)	0.16 ± 0.01 (0.17, 0.15~0.17)	0.18 ± 0.00 (0.19, 0.17~0.19)	**0.001**	**0.002**	0.771	0.697
VPI	0.39 ± 0.00 (0.39, 0.38~0.40)	0.39 ± 0.00 (0.39, 0.38~0.40)	0.36 ± 0.01 (0.35, 0.35~0.37)	0.41 ± 0.00 (0.41, 0.40~0.42)	0.36 ± 0.01 (0.36, 0.34~0.37)	0.35 ± 0.01 (0.37, 0.33~0.37)	0.35 ± 0.01 (0.36, 0.33~0.37)	0.39 ± 0.01 (0.41, 0.38~0.41)	**0.002**	**0.001**	0.816	0.631
VCI	485.9 ± 5.6 (485.3, 474.5~497.3)	642.9 ± 12.1 (632.9, 618.4~667.5)	471.9 ± 5.5 (472.5, 460.6~483.1)	592.8 ± 7.3 (599.0, 578.0~607.6)	439.0 ± 11.9 (455.1, 414.7~463.3)	587.8 ± 16.1 (603.9, 555.0~620.6)	431.0 ± 12.9 (454.6, 404.7~457.3)	567.4 ± 8.3 (577.0, 550.6~584.3)	**0.001**	**0.022**	**0.012**	**0.022**
VDI	17.90 ± 0.08 (17.85, 17.73~18.07)	18.04 ± 0.09 (18.11, 17.86~18.23)	17.86 ± 0.08 (17.84, 17.70~18.02)	18.08 ± 0.09 (18.11, 17.90~18.26)	17.84 ± 0.12 (17.72, 17.60~18.08)	17.93 ± 0.09 (17.81, 17.75~18.12)	18.02 ± 0.12 (17.82, 17.77~18.27)	17.97 ± 0.10 (17.97, 17.78~18.16)	0.422	0.212	0.787	0.360
Flux	0.48 ± 0.01 (0.50, 0.46~0.50)	0.46 ± 0.01 (0.45, 0.43~0.48)	0.50 ± 0.01 (0.51, 0.48~0.51)	0.47 ± 0.01 (0.46, 0.45~0.50)	0.46 ± 0.01 (0.45, 0.44~0.48)	0.42 ± 0.01 (0.42, 0.40~0.45)	0.48 ± 0.01 (0.50, 0.45~0.50)	0.45 ± 0.01 (0.45, 0.43~0.48)	0.306	**0.025**	0.428	0.379

*RNFL, retinal nerve fiber layer; VAD, vessel area density; VCI, vessel complexity density; VDI, vessel diameter index; VPI, vessel perimeter density; VSD, vessel skeleton density*.

*Data are presented as mean ± standard error (median, 95% confidence interval)*.

a *Mann-Whitney U test, P-value <0.05 was considered to be statistically significant. Significant values are shown in bold*.

Comparing ODD and control eyes *numerically*, in the superior and nasal quadrants, RNFL thickness was lower by 20 and 19%, respectively; VAD by 10 and 11%; VSD by 6 and 11%; VPI by 8 and 10%; and VCI by 10 and 9%. In the inferior quadrant, only two OCTA measurements (VAD by 9%, VCI by 9%) were significantly decreased compared with that of the controls. Despite 10% relatively lower RNFL thickness in the inferior quadrant in the ODD group, there was no significant different compared with that of control. In the temporal quadrant, only VCI was significantly decreased in ODD eyes (by 4%). Taken together, nasal and superior quadrants—where ODD were most commonly found—were most affected in ODD eyes, with significant thinning of RNFL thickness and significant reduction of at least four out of six OCTA measurements (VAD, VSD, VPI, VCI, and flux but not VDI) compared with controls (Table 2). Thus, VCI and VAD appeared to be the most useful measurements given a significant decrease in the ODD eyes compared with controls in at least three quadrants, while RNFL, VSD, and VPI were significantly decreased in the superior and nasal quadrants—the quadrants most commonly affected in ODD.

Despite these relatively greater decrements in peripapillary OCT and OCTA parameters in the superior and nasal quadrants, visual field MD (recalculated to reflect MD for each quadrant according to the modified Garway-Heath map; see section “Methods” and Figure 1) was the same in the four quadrants (*P* = 0.478). The average MD in each of the superior, nasal, and inferior quadrant in the ODD group were −3 dB, while the average MD in the temporal quadrant was −2 dB. Since ODD eyes are known to exhibit an asymmetric pattern of visual field defect localizing to the optic nerve head, this similarity of recalculated MD among four quadrants was unexpected. However, further analysis revealed that 68% of ODD eyes had MD better than −5 dB, and examination of the 32% of eyes with average MD equal or worse than −5 dB revealed that there was clear asymmetry of visual field MD with worse visual field (from worst to best) in the inferior > superior > temporal > nasal visual fields.

### Correlation of OCT, OCTA, and Corresponding Visual Field Mean Deviation in Quadrants

**Figure 2:**
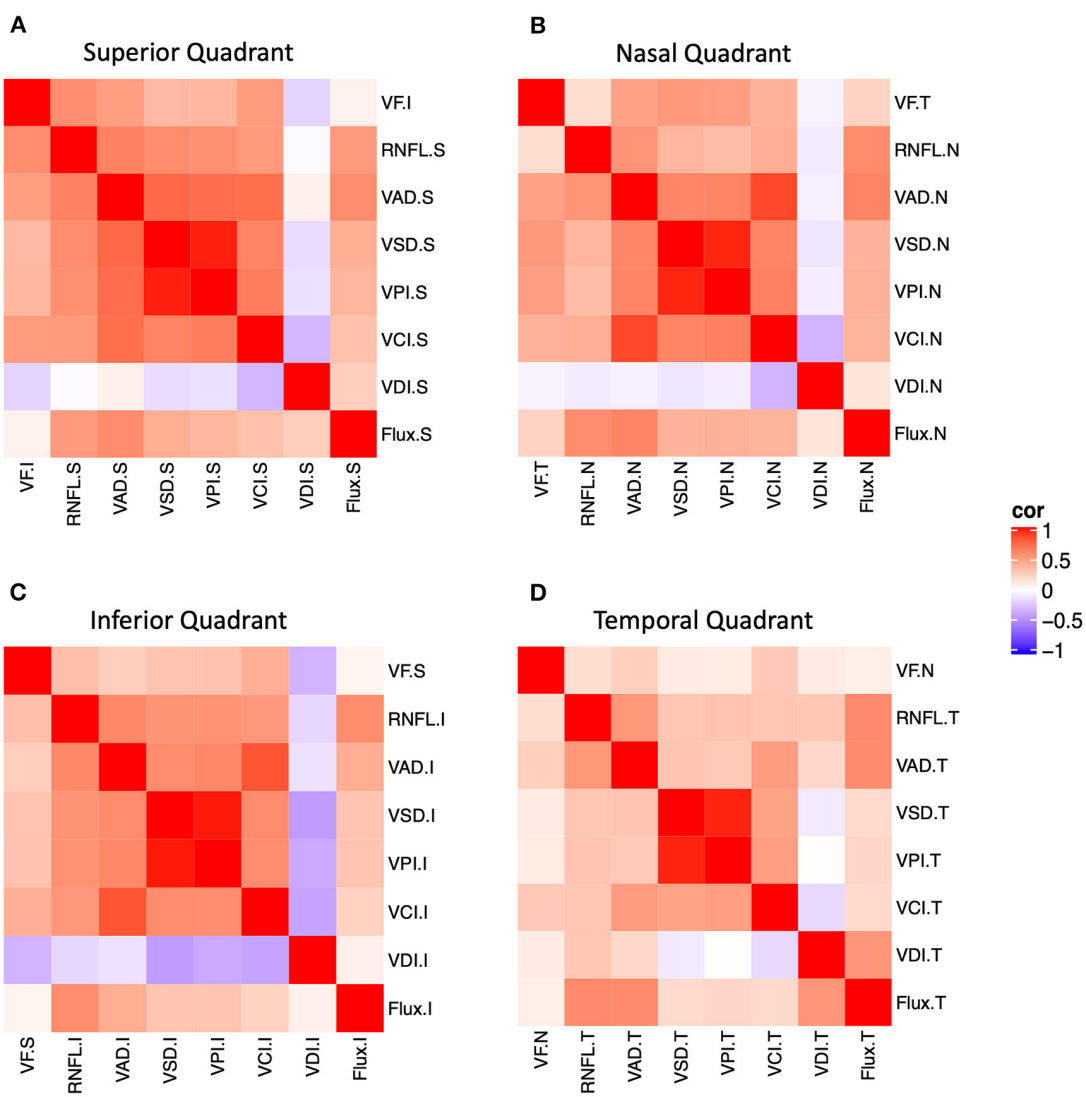
Structure-function and structure-structure correlation matrix heatmap of static perimetry mean deviation and corresponding quadrant measurements of OCT RNFL and six peripapillary OCTA parameters in **(A)** superior, **(B)** nasal, **(C)** inferior, and **(D)** temporal quadrants. Note OCT/OCTA *superior* quadrant corresponds to the *inferior* visual field. S, superior quadrant; T, temporal quadrant; I, inferior quadrant, N, nasal quadrant. VAD, vessel area density; VCI, vessel complexity index, VDI, vessel diameter index; VPI, vessel perimeter index; VSD, vessel skeleton density.

We performed structure-structure correlation of quadrant measurements of peripapillary OCT RNFL and five most useful OCTA measurements (OCTA VAD, VSD, VPI, and flux) (Figure 2 and Supplementary Figure 1). We found that, in general, OCTA measurements in the superior, nasal, and inferior quadrants were more strongly correlated with RNFL than that of the temporal quadrant. In the superior, inferior, and nasal quadrants, RNFL was moderately correlated with all five OCTA measurements (VAD, VCI, VSD, VPI, flux). In the temporal quadrant, only VAD, VPI, and flux were moderately correlated with RNFL.

We also performed structure-function correlation using visual field MD for each quadrant, OCT RNFL, and five OCTA measurements (VAD, VCI, VSD, VPI, flux). We found that MD was weakly or moderately correlated with RNFL and OCTA measurements (VAD, VCI, VSD, VPI) in the superior and inferior quadrants, relatively less in the nasal quadrant (VAD, VCI, VSD, VPI), and not in the temporal quadrant (Figure 2 and Supplementary Figure 1). For example, corresponding *inferior* visual field MD was highly and significantly correlated with *superior* quadrant RNFL, VAD, VCI, VSD, and VPI, and corresponding *temporal* visual field MD was highly correlated with *nasal* quadrant VAD, VCI, VSD, and VPI. Thus, quadrant analysis revealed that there was significant structure-function correlation in the superior and nasal quadrants, despite only a slight MD decrement in all quadrants.

## Discussion

*In vivo* ophthalmic imaging using OCT and OCTA provides valuable, non-invasive structural and vascular measurements of the optic nerve in patients with ODD. Given that ODD typically impact the nasal and superior optic disc disproportionately, we performed a detailed quadrant analysis of peripapillary OCT and OCTA measurements and corresponding visual field MD per Garway-Heath map in order to understand which measurements at each quadrant are most impacted in ODD. We found that OCTA *VAD* and *VCI* are significantly decreased in superior, nasal, and inferior quadrants in ODD eyes compared with controls, making them potentially the best OCTA measurements in control vs. ODD disease classification. OCT *RNFL* and two other OCTA measurements (*VSD, VPI*) are significantly affected in the superior and nasal quadrants—parts of the optic disc most affected in ODD. Despite these topographic differences in OCT RNFL and OCTA quadrants, corresponding visual field MD was similarly and mildly decreased because the majority of ODD eyes had relatively good visual function in our study. Subgroup analysis of eyes with average MD equal or worse than −5 dB revealed that the superior quadrant of the optic disc (inferior arcuate visual field) had the worst corresponding MD and that the temporal quadrant of the optic disc (includes the four paracentral points) had the best corresponding MD, consistent with the most common visual field pattern in ODD—altitudinal visual field defect with good central/paracentral vision. Our data show that in ODD-associated optic neuropathy, decrease in peripapillary OCT RNFL and OCTA can occur without parallel, measurable change in visual function. Our findings provide support to the diagnostic utility of performing peripapillary OCTA analysis in addition to OCT RNFL and suggest that future larger studies should investigate the sensitivity of *objective* measurements like OCT and OCTA since they may be more sensitive diagnostic indicators of optic neuropathy than *subjective* measurements like luminance-based perimetry.

OCTA is a novel technology that is increasingly utilized in assessment of patients with visual dysfunction due to neuro-ophthalmic diseases (14, 19). There has been anecdotal evidence that there is reduction in local peripapillary vessel density in peripapillary sections with a large volume of ODD (23). Our study provides the most detailed analysis to-date demonstrating the utility of this technology in patients with ODD. Using a custom algorithm, we were able to show that vessel density or VAD, the most common OCTA measurement, and VCI, which reflects the degree of the branching of perfused vasculature (27), were the most valuable potential predictors of vascular changes in ODD. Although we are the first to analyze quadrant VCI in ODD, a study in glaucoma, which was performed *with* large vessel removal, reported that VCI is a good diagnostic biomarker and significantly associated with structural and functional measures (27). Three other OCTA measurements—VPI, a measurement that reflects the perimeter of the vessels, VSD, which represents the vessel length density, and *flux*, which approximates blood flow intensity in vessel area (27)—are helpful in some quadrants in segregating ODD from that of controls. Given these three measurements all highly correlated with VAD and VCI, it may be sufficient to measure just VAD and VCI as the most useful diagnostic OCTA measurement in segregating ODD from control eyes.

Our study demonstrates the utility of peripapillary vessel area density as a valuable measurement, which has been previously reported in ODD and many other optic neuropathies (19, 27). Quadrant OCT and OCTA analysis is particularly important in ODD because ODD are most common in the superior and nasal quadrants in idiopathic ODD as well as syndromic ODD (2, 5), the ganglion cell axons are most likely to be affected in these areas. Consistent with this localization, we confirmed that OCT RNFL and most OCTA measurements were most affected in the superior and nasal quadrants. Gili et al. demonstrated that RNFL in all quadrants except temporal region of visible ODD was significant thinner than controls (17). In a study of OCTA quadrant analysis in 45 ODD eyes and 50 control eyes, which was performed using a 700-μm diameter annulus *without* large vessel removal, there was significant reduction of OCTA vessel density in all quadrants (31). Analysis of the entire peripapillary region or the hemi-superior or inferior peripapillary annulus *with* large vessel removal revealed that vessel density was significantly reduced in the ODD eyes compared with controls (31). Further quadrant analysis found the RNFL of superior sector in eyes with ODD showed a significant decrease compared with control group, while this reduction was not found in average RNFL (31). In another study of 10 ODD eyes and 10 control eyes, using quadrant OCTA measurements *without* large vessel removal, vessel density in the inferior nasal quadrant was significantly decreased compared with controls, although the average peripapillary vessel density was not significantly different between ODD and controls (32). Although we and others (31, 32) find that retinal VAD is a useful measurement in segregating ODD from controls in at least some quadrants or for the eye, a study including only pediatric patients did not (33). In a study of 18 pediatric ODD patients and 13 controls, which was performed using peripapillary OCTA measurements *without* large vessel removal, Alarcon-Toma et al. did not find any significant changes in retinal layers or in quadrants, although they did demonstrate significant changes in the choriocapillaris (33). Overall, we recommend using multiple peripapillary OCTA measurements, especially VAD and VCI in assessment of patients with ODD. Also, analysis of peripapillary OCTA should consistently only include small vessels (i.e., performed with large vessel removal), so only capillaries that reflect optic nerve perfusion are quantified.

Structure-structure and structure-function correlation analyses are useful for diagnosis and tracking the progression. We found that OCT RNFL and many OCTA quadrant measurements, especially VAD and flux, were significantly correlated with each other in quadrants. Engelke et al. found the RNFL had high significant positive correlation with peripapillary capillary vessel density (31). Some studies had demonstrated that correlation of visual function and OCT structure in ODD, that is, decreased RNFL was associated with visual field defect (7, 26). However, limited information is known about the quadrant correlation of structure and function parameters. Unlike OCT and OCTA measurements, where there was an obvious regional difference in measurement, with nasal and superior quadrants most affected, visual field MD for all quadrants were similarly and mildly affected. A larger study is needed to better understand this discrepancy, although our finding is compatible with that of the study by Malmqvist et al. used multifocal visual-evoked potential (mfVEP) amplitude to examine regional changes in visual field in 33 ODD patients and 22 controls. The hypothesis in their study is that mechanical compression of the unmyelinated retinal ganglion cell axons at the optic disc by ODD was the cause of visual field loss, so they predicted that optic nerve dysfunction caused by ODD affected mainly mfVEP amplitude. They found that there was significant correlation between mfVEP amplitude, perimetric mean deviation, and OCT RNFL thickness. Although there was a significant reduction of averaged mfVEP amplitude as well as the inner (from 0.87 to 5.67° radius) and outer rings (from 5.68 to 24° radius) compared with controls, there was an overall smaller amplitude of responses without obvious decrement corresponding to optic disc quadrants (34).

There are several limitations in this study. This is a small, retrospective study, and a larger, prospective study, ideally with serial visual field, OCT, and OCTA measurements, are needed, in order to provide strong evidence of the importance of quadrant analysis. Such study, performed with large vessel removal, can identify which of these OCT and OCTA measurements can be best diagnostic or prognostic biomarkers in future ODD clinical trials. Another caveat of our study is the age of the patients, since we primarily focused on the adult population. A study using quadrant analysis in the pediatric population can determine whether quadrant analysis is more sensitive to detect regional changes in evolving ODD patients, especially as ODD enlarge and migrate superficially with age. Another technical limitation is the differences in region of interest used to calculate peripapillary OCT RNFL and OCTA. OCT RNFL was segmented on a circular B-scan with 3.46 mm diameter centered on the optic disc, while OCTA of the superficial capillary plexus was measured using *en face* 3 × 3 images of an annulus with an outer diameter of 2.75 mm and an inner diameter of 1.5 mm centered around the disc (6 × 6 mm image are not as high resolution for vascular analysis). In addition, there are slight differences in the quadrant regions of interest between OCT and OCTA, since OCT RNFL quadrants were calculated using automatic segmentation and commercial software, while OCTA quadrants were divided according to the Garway-Heath map. While not exactly matching, these are reasonably good peripapillary OCT and OCTA measurements for quadrant analysis and comparisons. Although we did not systematically measure refractive error and axial length as contributing factors in this study, high myopic patients had been ruled out, and all the OCT B-scans indicated normal ocular axial length.

## Conclusions

Our study showed that ODD eyes exhibit particularly lower peripapillary RNFL thickness, VAD and VCI in the nasal and superior quadrants, consistent with the predominance of ODD in the nasal superior disc. Although this regional difference of OCT and OCTA measurements was thought to correspond to the progressive inferonasal and inferotemporal pattern of visual field defect, we found that there was similarly mild visual field MD in all quadrants per modified Garway-Heath map. Overall, many OCTA measurements highly correlated with VAD and VCI, so these two measurements are likely sufficient to segregate ODD from control eyes. Our data support a model of progression of changes in ODD, first affecting OCTA, then OCT, then visual field.

## Data Availability Statement

The raw data supporting the conclusions of this article will be made available by the authors, without undue reservation.

## Ethics Statement

The studies involving human participants were reviewed and approved by Institutional Review Board of Stanford University. Written informed consent to participate in this study was provided by the participants' legal guardian/next of kin.

## Author Contributions

YY and YL contributed to the conception or design of the work and made acquisition, analysis, and interpretation of data for the work. XZ, ZC, LS, MS, and RW contributed to the acquisition, analysis, and interpretation of data for the work. YY, XZ, ZC, LS, MS, RW, and YL drafted the manuscript, critically reviewed the manuscript for important intellectual content, approved the final version of the manuscript, and agreed to be accountable to all aspects of this work ensuring that questions related to the accuracy or integrity of any part of the work are appropriately investigated and resolved. All authors contributed to the article and approved the submitted version.

## Conflict of Interest

Although RW and ZC were not directly involved in data analysis in this study, they did have important disclosures related to OCTA analysis algorithm. RW has intellectual property owned by the Oregon Health and Science University and the University of Washington related to OCT angiography, which are licensed to commercial entities and related to the technology and analysis methods used in this manuscript. RW also receives research support from Carl Zeiss Meditec, Inc and Moptim Inc. RW is a consultant to Carl Zeiss Meditec, Inc. and Insight Photonic Solutions. ZC has intellectual property owned by the University of Washington related to OCT angiography, which are related to the technology and analysis methods described in this manuscript. The remaining authors declare that the research was conducted in the absence of any commercial or financial relationships that could be construed as a potential conflict of interest.

## Supplementary Material

Supplementary Figure 1 The correlation coefficients and p values of correlation matrix heatmap shown in Figure 2, which demonstrate the correlation of static perimetry mean deviation and quadrant measurements of OCT RNFL and six OCTA parameters in **(A)** superior **(B)** nasal, **(C)** inferior, and **(D)** temporal quadrants. The correlation coefficients between 0 and 0.3 indicate weak correlation, between 0.3 and 0.7 indicate moderate correlation, and between 0.7 and 1 indicate high correlation. Asterisk, the correlation is statistically significant. S, superior quadrant; T, temporal quadrant; I, inferior quadrant; N, nasal quadrant; VAD, vessel area density; VCI, vessel complexity index; VDI, vessel diameter index; VPI, vessel perimeter index; VSD, vessel skeleton density.

## Abbreviations

BCVA: best corrected visual
EDI-OCT: enhanced-depth OCT
MD: mean deviation
OCT: optical coherence tomography
OCTA: OCT angiography
ODD: optic disc drusen
VAD: vessel area density
VCI: vessel complexity index
VDI: vessel diameter index
VPI: vessel perimeter index
VSD: vessel skeleton density.
