# Supplementary figures and images for: Topographic Quadrant Analysis of Peripapillary Superficial Microvasculature in Optic Disc Drusen
Source: Front Neurol. 2021 May 19;12:666359. doi: 10.3389/fneur.2021.666359 (PMC8170317; doi:10.3389/fneur.2021.666359)

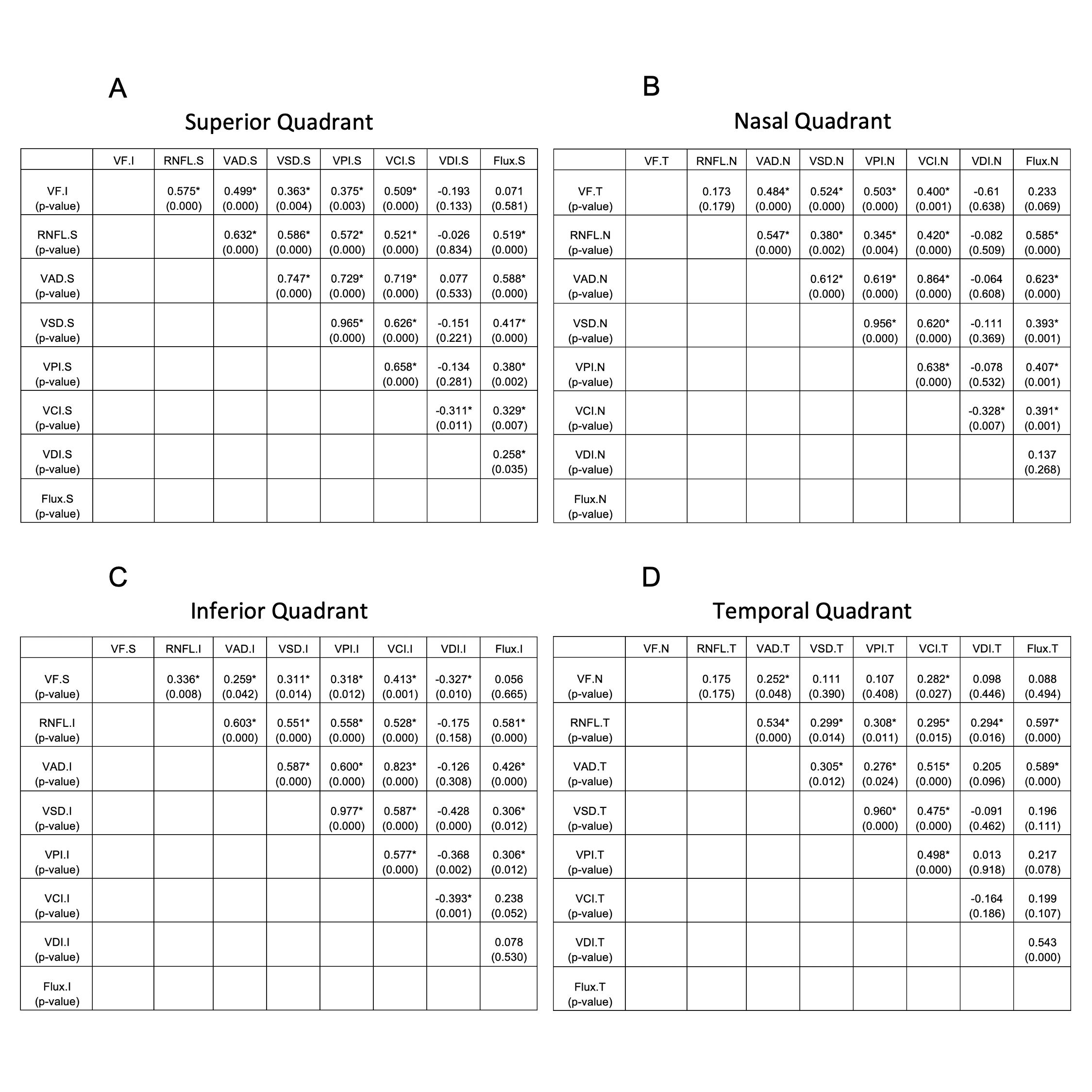

Supplement: Supplementary Figure 1 — The correlation coefficients and p values of correlation matrix heatmap shown in Figure 2, which demonstrate the correlation of static perimetry mean deviation and quadrant measurements of OCT RNFL and six OCTA parameters in (A) superior (B) nasal, (C) inferior, and (D) temporal quadrants. The correlation coefficients between 0 and 0.3 indicate weak correlation, between 0.3 and 0.7 indicate moderate correlation, and between 0.7 and 1 indicate high correlation. Asterisk, the correlation is statistically significant. S, superior quadrant; T, temporal quadrant; I, inferior quadrant; N, nasal quadrant; VAD, vessel area density; VCI, vessel complexity index; VDI, vessel diameter index; VPI, vessel perimeter index; VSD, vessel skeleton density. [file Image_1.TIFF]
